# An Urgent Problem

**Published:** 1920-03-20

**Authors:** 


### An Urgent Problem.

THE very definite relation of pure milk to the prosperity of the young was strikingly illustrated at the conference of the Scottish Federation of Mother and Child-Welfare Associations. Mr. John Fraser, M.D., F.R.C.S., addressed the conference on the dangers of impure milk, and said perhaps in some ways it was a pity that milk was the food *par excellence* which a child required. It was a warm fluid, full of nourishment; but in a fluid of that nature poisons flourished. The results were not seen a few weeks after the poison had got into the child's system. They might appear months, or even years, later. The disasters following on an impure milk supply were, from the surgical point of view, almost limited to the question of tuberculosis. In the children's hospitals, it was astonishing to see the number of children in the surgical beds, 30 or 40 per cent., who were infected with tuberculosis. The child was given tuberculous milk. These poisons settled in various parts of the child's digestive system. Some settled in the tonsils, and so into the glands of the neck—a disease which was extraordinarily prevalent. That was not a very dangerous type.

It was the other type which was so insidious and so dangerous—when the milk had passed lower down into the child's digestive system, and had settled down in the intestines. Tuberculosis passed into the blood, and the child suffered from tuberculous blood poison, which circulated all through its body, and began to settle down in various parts. It settled in the bones and joints, a most painful, serious, and disastrous disease. A child who had this disease in early life was continuously handicapped afterwards. The question of tuberculosis was all traceable to an imperfect milk supply; and if they could do anything to lessen the dangers to which he had referred, their work would be well done.

Mr. Fraser answered a number of questions which were asked at the conclusion of the address. In answer to the question why milk was so largely advocated if it was so dangerous, Mr. Fraser said they had nothing better to give children; and with precaution it could be rendered harmless. During the last three years there was no doubt that tuberculosis had increased tremendously. They believed that the reason was the diminished supply of fats, which formed one of the strongest resistive powers that the child had. In answer to another question as to why the mortality amongst West End children was not heavier than in the East End, where milk was less used, Mr. Fraser said the nutrition of the children in the West End was very much better, and so they had a greater degree of resisting power than the children in the East End. With regard to natural feeding, human milk tended to deteriorate after six or seven months, and should not be continued for longer than nine months.

Dr. Alexander Lauder, D.Sc., who described the present condition of milk supply as extremely unsatisfactory, declared that some centralisation of distribution was absolutely necessary; the control need not necessarily be municipal, as it could be in the hands of one or two large firms. So long as milk was sold by all sorts of people in little shops, no real control was possible, and centralisation was to be advocated both on grounds of economy and because control would be made much easier.

The conference decided to assist so far as it could in the formation of a branch of the National Clean Milk Society, and to urge the Government to reintroduce the Tuberculosis Order and proceed with the Milk and Dairies Act amendment so that its regulations might be brought into force as soon as possible.

